# Pulmonary interstitial emphysema in fatal asthma: case report and histopathological review

**DOI:** 10.1186/s12890-018-0615-7

**Published:** 2018-03-20

**Authors:** Thais Mauad, Felipe B. P. do Nascimento, Marisa Dolhnikoff, Milena C. M. Picka, Paulo H. N. Saldiva, on behalf of BIAS

**Affiliations:** 1 Department of Pathology, São Paulo University Medical School, São Paulo, Brazil; 2 Radiology Institute, Hospital das Clínicas, São Paulo University Medical School, São Paulo, Brazil; 3 Faculdade de Medicina da Universidade de São Paulo, Av. Dr. Arnaldo, 455, sala 1155, 01246- 903, São Paulo, Brazil

**Keywords:** Fatal asthma, Autopsy, Post-mortem computerized tomography, Pulmonary interstitial emphysema

## Abstract

**Background:**

Mortality related to asthma has decreased worldwide since the introduction of inhaled corticosteroid therapy in the past decades. However, there are still some asthma fatalities identified mainly in populations with less access to regular treatment. Pulmonary interstitial emphysema due to alveolar rupture has been rarely described as a complication of an acute severe asthma attack, and its identification in pathological analysis can be difficult. Previous studies reported the association of pulmonary interstitial emphysema and bronchial ductal gland ectasia in asthma.

**Case presentation:**

We present the case of a 42-year- old man that died due to a fatal asthma attack. Postmortem computed tomography revealed the unusual finding of acute Pulmonary Interstitial Emphysema, confirmed by pathological analysis. We reviewed 28 cases of fatal asthma tissue and identified the presence of pulmonary interstitial emphysema in 10% of the cases.

**Conclusions:**

Postmortem computed tomography is a useful complimentary diagnostic tool for autopsies. Pulmonary Interstitial Emphysema in acute exacerbations of asthma seems to be more frequent than reported. Alveolar hyperdistension and bronchial duct gland ectasia causing tissue rupture are possible mechanisms involved in the formation of Pulmonary Interstitial Emphysema. The clinical impact of Pulmonary Interstitial Emphysema in asthma is unknown.

## Background

Asthma affects more than 300 million people worldwide being a considerable health burden [1]. Between 1990 and 2013, advances in asthma management resulted in a 42% reduction in age-standardized death rates worldwide [2]. Decline in asthma mortality has been also observed in Brazil since the late 90s, when inhaled corticosteroid therapy became universally available. Inequalities still persist in the country; the mortality decreased more significantly in the Southeast region of the country [3].

Death due to asthma is believed to be a combination of airway smooth muscle bronchoconstriction and asphyxia due to mucus plugging. The lungs of patients who have died of asthma are usually hyperinflated due to the persistently increased intrapulmonary pressure. Macroscopically, except for bronchial thickening and mucus plugs there are no other prominent alveolar parenchymal changes [4].

Pulmonary Interstitial emphysema (PIE) is an unusual condition wherein air dissects through alveolar walls into the adjacent interstitial tissues. It has been described in premature babies, blunt thoracic trauma, mechanic ventilation, lung obstructive diseases, infectious diseases and idiopathic pulmonary fibrosis. When associated with pneumomediastinum, this process is known as Macklin effect [5–7]. In living asthmatics, there are few descriptions of PIE as a complication of persistent disease [8] and exacerbations [9]. Cluroe et al., however, described that 20% of fatal asthma cases in New Zealand in the late 80’s had histological signs of PIE, associated with bronchial gland duct ectasia. These authors suggested that duct rupture due to mucus accumulation could cause air entry into the interstitium [10, 11]. This finding was not associated to any specific clinical feature related to the final attack, such as interval between death and autopsy [12]. Other authors did not report PIE when studying pathological findings in fatal asthma [13–15].

Here, we describe a case of fatal asthma where PIE was diagnosed by post-mortem computed tomography of the thorax (PMCT) and later confirmed by histology. In addition, we reviewed 28 cases of fatal asthma from our autopsy files to describe the percentage of cases with histological features of PIE that were possibly left undiagnosed. We also discuss possible mechanisms for the formation of PIE in fatal asthma.

## Case presentation

A forty-two-year-old man was found dead at home and referred to our coroner service. Interview with the next-of-kin prior to autopsy revealed that the patient had non-treated asthma, hypertension and a non-specified psychiatric disease. In the previous days before death, the patient had episodes of wheezing, but did not receive any specific asthma treatment. There was no suspicion of exogenous intoxication. The next-of-kin provided written consent authorizing autopsy results for research purposes.

**Fig. 1:**
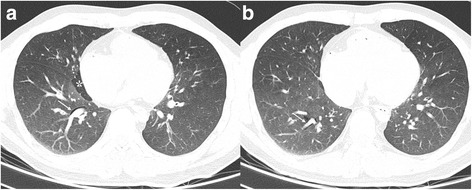
Post mortem computed tomography. High-resolution lung images show distended lungs with extensive bilateral air trapping, sometimes heterogeneous as pointed in *, associated with a decrease in vascularity. PIE dissects peribronchovascular interstitium in the right lower lobe, besides segmentary (**a**) and subsegmentary (**b**) bronchi. There is also bronchial wall thickening with associated narrowing of bronchial lumen.

PMCT prior to autopsy (performed 13hs after death) revealed diffusely constricted airways, with wall thickening and mucus impaction. There were signs of air trapping, characterized by mosaic lung attenuation, pulmonary hyperinflation, and a focal, centrally localized interstitial emphysema (Fig. 1).

Autopsy revealed a eutrophic man, without external signs of violence. There was no subcutaneous emphysema or signs of pneumomediastinum. Both lungs were markedly hyperinflated and did not collapse with the opening of the thorax cavity. There was no pleural effusion and the pleural surfaces had a normal aspect. There were moderate mucus plugs within the airways and no parenchyma abnormalities. Other organs had no pathological abnormalities, except for extreme congestion.

**Fig. 2:**
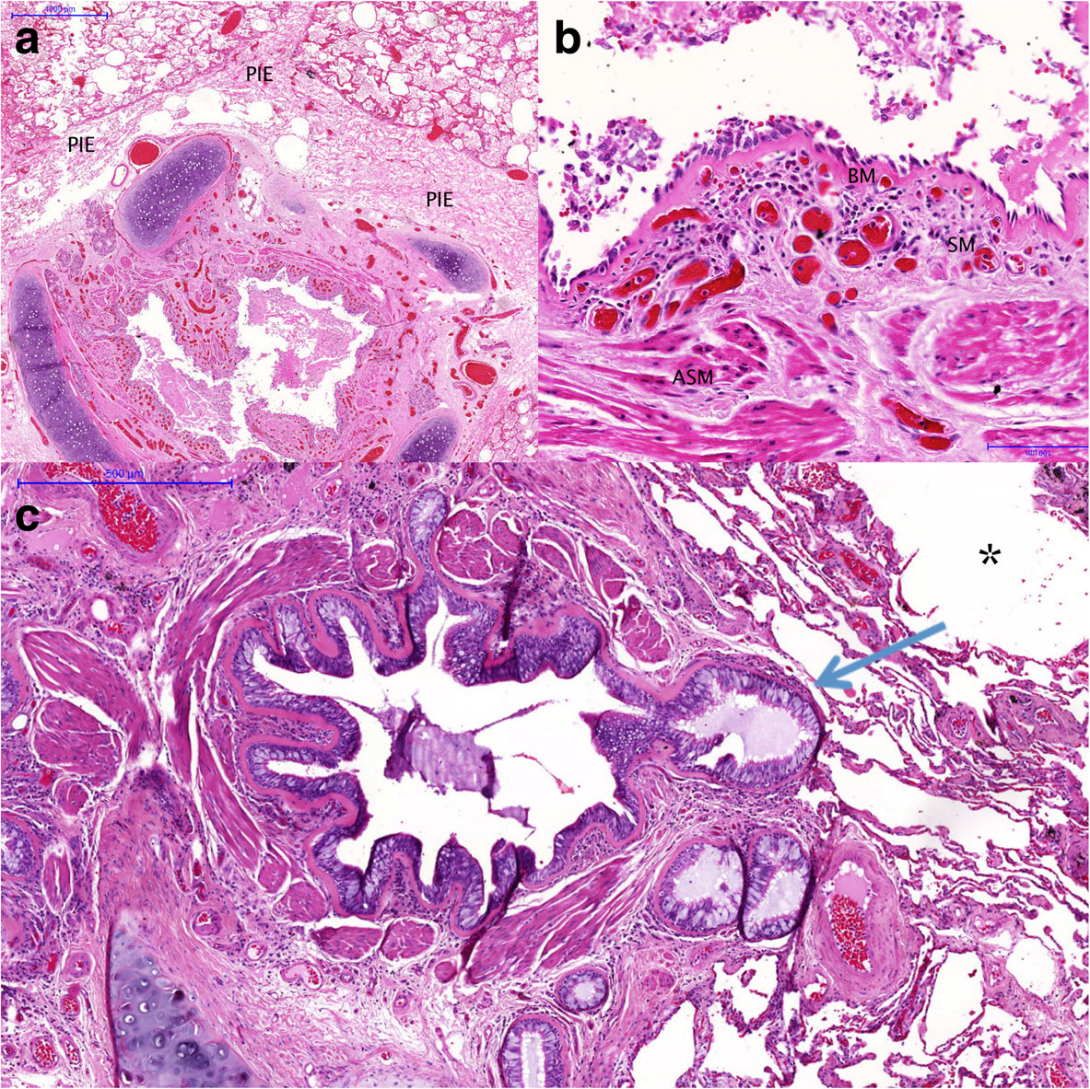
**a** Constricted large airway, with thickened airway smooth muscle, mucosal congestion and mucus plugging. Observe the widened bronchovascular bundles, with tearing of the connective tissue. **b** Detail of a large airway showing thickened basement membrane, capillary ectasia and congestion, eosinophilic inflammation and a thick airway smooth muscle bundle, consistent with asthma. **c** Lung section of a fatal asthma case showing bronchial duct gland ectasia with chronic inflammation, common findings in fatal asthma. PIE - Pulmonary Interstitial Emphysema; BM = Basement Membrane; SM = Submucosa; ASM = Airway Smooth Muscle; * = alveolar hyperdistension, arrow = bronchial duct ectasia

Microscopic analysis of the lungs showed a histological picture typical of asthma: Medium to large bronchi had hyaline and thickened basement membrane, airway smooth muscle thickening, eosinophilic inflammation and capillary ectasia in the submucosa (Fig. 2a and b). Airway lumens were partially filled with mucus exudates. There was a widening of peribronchial and perivascular regions, with tearing of the connective tissue, consistent with interstitial emphysema (Fig. 2a). We observed the presence of bronchial gland duct ectasia, but no signs of gland rupture (Fig. 2c). There were signs of alveolar tear due to hyperdistension.

### Histological case-series

It can be difficult to differentiate PIE from peribronchial and septal edema, a common finding in autopsies. Therefore, we reviewed 28 cases of fatal asthma from our archives files in order to retrospectively check which cases would fulfil the criteria previously described by Cluroe et al. [8]: Interstitial emphysema was considered when there was wide disruption and tearing of peribronchial and perivascular connective tissue. If there was uncertainty in distinguishing interstitial emphysema from sectioning artefact. Interstitial emphysema was not scored as being present. The use of these cases for research purposes has been previously approved by our local ethical board (approval number 360/12). To guarantee adequate sampling, we selected the cases with more than 10 sections available for analysis. Asthmatics had a median age of 49 years (range 20–71 years), 15 were female, 15 were non-smokers and their asthma duration had a mean of 32 years. The fatal crisis had a duration of <2hs in 2 subjects, between 2-24hs in 9, >24hs in 15 and was unknown in 2. Twenty-seven of the cases had bronchial duct gland ectasia and all had alveolar hyperdistension. No gland duct rupture was found (Fig. 2c). We found 3 cases with a histological diagnosis of PIE that were not diagnosed clinically. They were 2 males and 1 female, 1 smoker/2 non-smokers and 1 had the duration of the last crisis between 2-24hs and 2 more than 24 h. All presented alveolar hyperdistention and gland duct ectasia.

## Discussion and conclusions

Here we show a rarely reported complication of a fatal asthma attack that is the presence of pulmonary interstitial emphysema, diagnosed by PMCT scan of the thorax. In addition, histological review of 28 cases of fatal asthma, revealed that this complication was present in 10% of the cases.

PMCT has been increasingly used for forensic purposes in recent years [16]. In hospitalized patients, PMCT has shown to be more useful than clinical diagnosis for identifying the immediate cause of death [17].The use of lung PMCT has been subject of research, because the degree of aeration and liquid distribution is obviously different than of breathing patients [18, 19]. Here we report that PMCT of the lungs is useful to diagnose PIE, a complication of asthma that also occurs in premature babies, blunt thoracic trauma, mechanic ventilation, lung obstructive diseases, infectious diseases among others [5–7].

CT scans of patients with asthma frequently show bronchial and bronchiolar wall thickening, airway lumen narrowing, small centrolobular opacities, air trapping identified as areas of decreased pulmonary attenuation, hyperinflation, emphysema and, rarely, cysts [20]. Interstitial emphysema has been previously reported as a rare finding in asthma, located adjacent to bronchi of first, second or third order [8, 9]. To our knowledge, this is the first report of the use of complimentary PMCT in fatal asthma that in addition to airway changes revealed PIE. PMCT is a useful complement to conventional autopsies.

The pathogenesis of PIE is usually attributed to the access of air to the lung interstitium, via either rupture of airspaces at the lobular periphery or rupture of overdistended small bronchioles, mainly during mechanical ventilation (also known as Macklin effect). It then dissects along the connective tissue sheets of bronchovascular bundles, veins and lymphatics of interlobular septa towards the pleural surface or the hilum [5–7]. Such phenomenon might alleviate the increased intrapulmonary pressure observed in an asthma attack. Cluroe et al. proposed an alternative method to explain spontaneous PIE in asthma [10]: In fatal asthma, ectatic and inflamed bronchial glands are not uncommon [11], and these authors believed that rupture of dilated bronchial gland ducts would occur due to increased pressure in the duct wall, leading to air entry and tearing of the bronchovascular connective tissues [10]. We believe that their theory is plausible and further speculate that excessive airway smooth muscle constriction and the presence of tenacious mucous plug could cause increased pressure at the gland wall. We have previously described increase in the contractile myoepithelial cells of the bronchial glands in fatal asthma, which could further increase the duct pressure [21]. In line with their hypothesis, PIE observed in this case had a central rather than peripheral localization in the lungs, as previously described by Demura et al. in patient with asthma misdiagnosed as emphysema [8]. In the present case, we found dilated bronchial gland ducts, but no signs of rupture. We cannot exclude PIE due to alveolar rupture since alveolar hyperdistention was present in this case, as in most cases of fatal asthma.

The extent to which PIE contributes to worsening of an asthma episode is not known. Cluroe et al. first hypothesized that such condition could be associated with a precipitous, sudden asthma attack, but could not relate PIE to any clinical condition, including length of the last asthma attack [12]. In our cases review, we found 10% of reviewed cases had histological diagnosis of PIE. Since we had a very limited sample (*n* = 3), we could not identify predictors of PIE or advance in its clinical significance. All the 3 cases reviewed presented alveolar hyperdistension, and the majority bronchial duct gland ectasia. We emphasize that pathologists should be aware about this diagnosis that can be overlooked due to its similarity to peribronchial or interlobular septal edema or confounded with sectioning artifacts. Accordingly, Jabra et al. studying lung explants showed that PIE was not cited in pathology reports, although present in 30% of the cases of usual interstitial pneumonia after histological revision [22].

In summary, we report the finding of acute PIE in fatal asthma using PMCT. PIE can be overlooked in histological sections but may occur in more than 10% of fatal asthma cases. The impact of PIE contributing to asthma death is unknown.

## Abbreviations

PIE: Pulmonary Interstitial emphysema
PMCT: Post-mortem Computed Tomography of the Thorax
